# Systematic Identification and Bioinformatic Analysis of MicroRNAs in Response to Infections of Coxsackievirus A16 and Enterovirus 71

**DOI:** 10.1155/2016/4302470

**Published:** 2016-10-24

**Authors:** Zheng Zhu, Yuhua Qi, Huan Fan, Lunbiao Cui, Zhiyang Shi

**Affiliations:** Key Laboratory of Enteric Pathogenic Microbiology, Ministry of Health, Jiangsu Provincial Center for Disease Prevention and Control, No. 172 Jiangsu Road, Nanjing 210009, China

## Abstract

Hand, foot, and mouth disease (HFMD), mainly caused by coxsackievirus A16 (CVA16) and enterovirus 71 (EV71) infections, remains a serious public health issue with thousands of newly diagnostic cases each year since 2008 in China. The mechanisms underlying viral infection, however, are elusive to date. In the present study, we systematically investigated the host cellular microRNA (miRNA) expression patterns in response to CVA16 and EV71 infections. Through microarray examination, 27 miRNAs (15 upregulated and 12 downregulated) were found to be coassociated with the replication process of two viruses, while the expression levels of 15 and 5 miRNAs were significantly changed in CVA16- and EV71-infected cells, respectively. A great number of target genes of 27 common differentially expressed miRNAs were predicted by combined use of two computational target prediction algorithms, TargetScan and MiRanda. Comprehensive bioinformatic analysis of target genes in GO categories and KEGG pathways indicated the involvement of diverse biological functions and signaling pathways during viral infection. These results provide an overview of the roles of miRNAs in virus-host interaction, which will contribute to further understanding of HFMD pathological mechanisms.

## 1. Introduction

Hand, foot, and mouth disease (HFMD) is a common illness among infants and young children, typically characterized by several days of fever, ulcerative vesicles in the oral mucosa, and maculo- or papulovesicular lesions on the hands, feet, and buttocks [1]. Coxsackievirus A16 (CVA16) and enterovirus 71 (EV71), which belong to the genus* Enterovirus* in the family Picornaviridae, are the two major pathogens causing HFMD. Although most HFMD cases present with mild and self-limiting clinical symptoms, a minority of patients, especially those infected with EV71 virus, rapidly develop severe neurologic complications such as encephalitis, aseptic meningitis, and acute flaccid paralysis, which can lead to pulmonary edema (PE) and even death. Furthermore, these neurologic complications may be also associated with neurologic sequelae, delayed neurodevelopment, and reduced cognitive functioning in children [2]. The outbreaks of HFMD have been reported in many places of the world including the United States [3], Germany [4], Australia [5], Malaysia [6], Taiwan [7], Singapore [8], and Brunei [9]. In the year 2008, large nationwide HFMD epidemic occurred in Mainland China with a substantial morbidity and mortality rate. A national enhanced surveillance system for HFMD has been therefore established in China to facilitate the epidemiological investigation of the disease [10]. There were 1 619 706, 2 168 737, and 1 828 377 cases in the whole country with 509, 567, and 252 deaths in the year of 2011, 2012, and 2013, respectively, reported by National Health and Family Planning Commission of the People's Republic of China. Thus far, no effective vaccines or antiviral drugs are available for HFMD and the molecular mechanisms underlying the infection of CVA16 and EV71 remain elusive. HFMD, therefore, has become a severe public health issue throughout the world.

MicroRNAs (miRNAs) are a class of small, noncoding RNA molecules with a length of 18~25 nucleotides and function as major gene expression regulators at the posttranscriptional level. Generally, these endogenous RNAs specifically target the 3′ untranslated regions (3′ UTR) of the mRNAs to result in mRNA degradation or translation inhibition based on the degree of sequence complementarity [11]. It has been estimated that more than one-third of all protein-coding genes seem to be miRNA targets in humans by conserved seed pairing [12]. A growing body of evidence has revealed that miRNAs are involved in diverse physiological processes, such as development [13], cell proliferation and differentiation [14, 15], apoptosis [16], and a variety of pathological conditions [17, 18]. Similarly, recent studies have also focused on the involvement of miRNAs in virus-host interaction networks. Latent infections with some viruses were demonstrated to alter the host cellular miRNA expression patterns, which might be tightly associated with the initiation and progression of diseases [19, 20]. It has been demonstrated that some cellular miRNAs could directly affect the virus replication. Human liver-specific miR-122 could be utilized to promote viral RNA replication of hepatitis C virus (HCV) [21]. On the other hand, overexpression of miR-30e^*∗*^ upregulated IFN-*β* and the downstream IFN-stimulated genes including OAS1, MxA, and IFITM1 to inhibit dengue virus (DENV) replication [22]. Hepatitis B virus (HBV) infection led to the alteration of miRNA expression profile in mouse and human hepatocytes with the upregulation of miR-486-3p, miR-1908, miR-675, and miR-1231, among which miR-1231 was able to suppress HBV replication by targeting core mRNA [23]. Studying miRNA-mediated virus-host interactions might therefore contribute to an elucidation of virus infection mechanism and potential identification of antiviral targets.

Previously there were also several studies reporting the role of miRNAs in enterovirus infection. Tang et al. identified that the expression of host cellular miR-197 was significantly downregulated by EV71 infection and then revealed a novel molecular mechanism that EV71-induced miR-197 downregulation could sustain host RAN protein level to facilitate viral replication [24]. A most recent study characterized the responses of serum miRNA profiles to various EV71 infection diseases and demonstrated that elevated expression of circulating miR-876-5p is a specific marker of severe EV71 infection [25]. Additionally, miR-432^*∗*^ was identified with the ability to stimulate the replication of CVA16 virus in host cells [26]. Although some small RNA molecules were revealed to be involved in the infectious cycle of CVA16 or EV71 by different researchers, no study, to date, was performed to systematically compare the common and differential aspects of cellular miRNA alterations and analyze related biological pathways upon two enteroviral infections. In the present study, we examined the expression profiles of host cellular miRNA in response to CVA16 and EV71 infections and identified miRNAs tightly related to replication process of two viruses. Meanwhile, target gene prediction and subsequent bioinformatic analysis enhanced our understanding of the roles of miRNAs in HFMD pathogenesis.

## 2. Materials and Methods

### 2.1. Cells and Viruses

Human RD (rhabdomyosarcoma) cells were cultured in Dulbecco's modified Eagle's medium (DMEM) (Gibco/Invitrogen, Carlsbad, CA) containing 10% fetal bovine serum (FBS) (Gibco), supplemented with 100 IU mL^−1^ penicillin G and 100 *μ*g mL^−1^ streptomycin (Gibco), and incubated at 37°C under a humidified atmosphere with 5% CO_2_. CVA16 and EV71 strains were isolated from the throat swab specimens of HFMD patients during an outbreak in 2008 in Jiangsu, China. The virus titer was determined by a plaque assay as described previously [27]. The stocks were stored in aliquots at −70°C.

### 2.2. Virus Infection Assay

RD cells were seeded in 6-well plates at a density of 1 × 10^6^ per well. After overnight incubation, cells were infected with CVA16 or EV71 virus at a multiplicity of infection (MOI) of 1. After absorption for 1 h at 37°C, the cells were washed twice with PBS and covered with DMEM medium containing 2% FBS for further culture at 37°C. The infected and control cells were harvested at 6 h after infection (hpi) and subjected to microarray or quantitative RT-PCR analysis.

### 2.3. miRNA Microarray

Total RNA extraction was performed from virus-infected cells and mock-infected cells using the Trizol reagent (Invitrogen) according to the manufacturer's instructions. The quality of purified RNA was assessed by agarose gel electrophoresis and spectrophotometer methods. miRNA expression profile was examined based on a microarray assay using a *μ*Paraflo™ microfluidic chip (LC Sciences, Houston, TX, USA) consisting of hybridization probes complementary to all human miRNAs listed in Sanger Institute miRBase Release 17.0. Data derived from the microarray were analyzed by subtracting the background fluorescence and then normalizing the signals using the locally weighted regression (LOWESS) method [28]. The ratios of normalized signals between virus-infected cells and control cells were calculated. Those miRNAs with a ≥1.5-fold change in expression were shown.

### 2.4. Validation of miRNA Expression Levels by Quantitative RT-PCR

miRNAs were isolated from virus-infected cells and control cells using miRNeasy Mini Kit (Qiagen, Germany) and subsequently reverse-transcribed (RT) into cDNA using the One-Step PrimeScript miRNA cDNA Synthesis Kit (TaKaRa, Dalian, China) following the manufacturer's instructions. Real time PCR assays were performed to determine the expression level of each miRNA with the use of SYBR* Premix Ex Taq* II Kit (TaKaRa). The assay was carried out in a 20 *μ*L reaction mixture containing 10 *μ*L of 2x SYBR* Premix Ex Taq* II, 0.4 *μ*L of ROX Reference Dye, 0.4 *μ*M of specific miRNA forward primer, 0.4 *μ*M of Uni-miR qPCR Primer (TaKaRa), and 2 *μ*L of diluted cDNA (1 : 50). The thermal cycling conditions were as follows: an initial denaturation step at 95°C for 30 s, 40 cycles of PCR amplification at 95°C for 5 s, and 60°C for 30 s, followed by a melting curve analysis program according to the instrument documentation. All real time PCR reactions were run in triplicate. U6 small RNA was used as an internal control. Fold change of each miRNA expression was calculated using the equation 2^−ΔΔCt^. The sequences of all the primers used were listed in Table 1.

### 2.5. Target Gene Prediction of Differentially Expressed miRNAs

Two computational target prediction algorithms, TargetScan (http://www.targetscan.org/) and MiRanda (http://www.microrna.org/), were used to predict the potential targets of the differentially expressed miRNAs, respectively. The obtained data were combined and the target genes predicted simultaneously by both algorithms were presented.

### 2.6. GO and KEGG Pathway Enrichment Analysis

GO and KEGG pathway enrichment analyses of miRNA target genes were performed as previously described [29]. For GO analysis, all of the target genes were matched to GO terms in the database (ftp://ftp.ncbi.nih.gov/gene/DATA/gene2go.gz) according to three categories: biological process, cellular component, and molecular function, and then the gene numbers for each term were calculated. The significantly enriched GO terms were obtained by comparing with the reference gene background with the use of hypergeometric tests. The calculating formula is as follows:

(1) $$\begin{array}{c} P=1-\overset{m-1}{\underset{i=0}{\sum }}\frac{\left(\begin{array}{c} M\\ i \end{array}\right)\left(\begin{array}{c} N-M\\ n-i \end{array}\right)}{\left(\begin{array}{c} N\\ n \end{array}\right)}. \end{array}$$

In this formula, *N* represented the number of GO annotated genes in whole genome, *n* represented the number of target genes in *N*, *M* was the number of single GO annotated genes in whole genome, and *m* was the number of target genes in *M*. Those GO terms with *P* < 0.01 were considered as significant enrichment.

Similarly, all of the target genes of differentially expressed miRNAs were annotated with corresponding KEGG pathways using the database (http://www.genome.jp/kegg/). Enrichment analysis was then performed using the same formula as GO, where *N* represented the number of KEGG annotated genes in whole genome, *n* represented the number of target genes in *N*, *M* was the number of genes with single KEGG pathway in whole genome, and *m* was the number of target genes in *M*. *P* < 0.01 was considered as significant enriched KEGG pathway.

## 3. Results

### 3.1. miRNA Expression Profiles in CVA16- and EV71-Infected Cells

**Figure 1:**
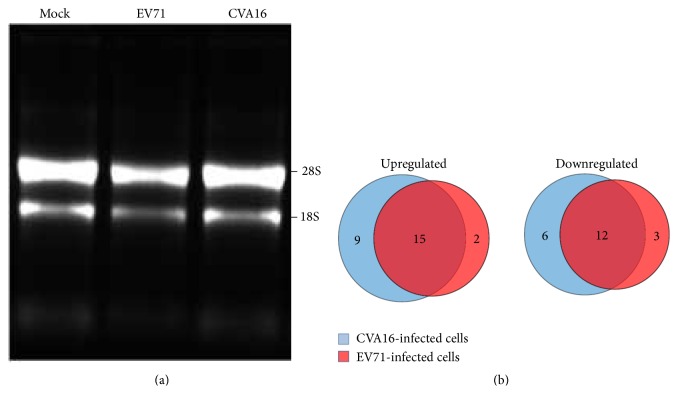
(a) Agarose gel electrophoresis analysis of total RNA purified from mock-infected and virus-infected RD cells. (b) The alteration of miRNA expression profiles in response to CVA16 and EV71 infection based on microarray analysis.

In order to investigate host cellular miRNA expression alterations upon CVA16 and EV71 infections, total RNA was extracted from mock-infected and virus-infected RD cells. Agarose gel electrophoresis analysis showed that the purified total RNA possessed good integrity in three samples (Figure 1(a)). Small RNAs were enriched using a YM-100 Microcon centrifugal filter and then subjected to miRNA microarray analysis. As shown in Figure 1(b), CVA16 and EV71 infections increased the expression levels of 26 cellular miRNAs compared with control cells, among which 15 miRNAs were commonly upregulated by both viruses with 9 CVA16-specific upregulation and 2 EV71-specific upregulation. Meanwhile, the expression levels of 12 miRNAs were decreased simultaneously by both viruses. 6 and 3 miRNAs were specifically downregulated by CVA16 and EV71 infections, respectively. The regulated miRNAs with fold changes were in detail listed in Table 2.

### 3.2. Validation of miRNA Expression Levels by Quantitative RT-PCR

**Figure 2:**
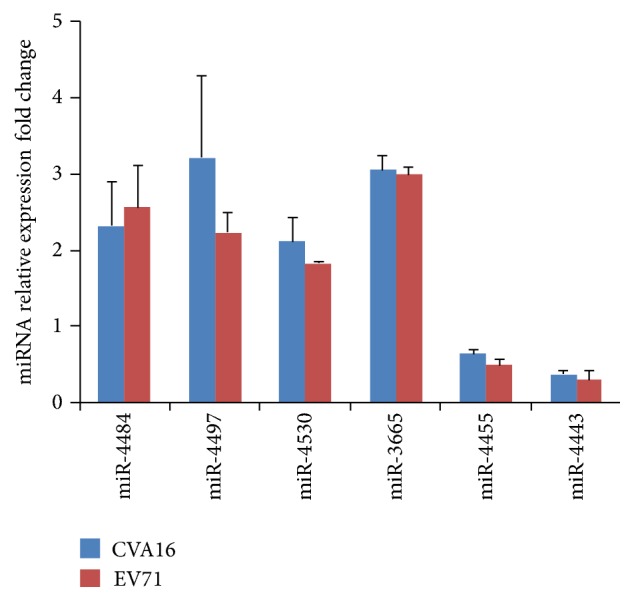
Quantitative RT-PCR was performed to examine the expression of miRNAs in virus-infected cells. The expression levels were normalized to endogenous U6 small RNA. Fold change compared with control cells was calculated using the equation 2^−ΔΔCt^. The data was presented as mean ± SD. Six miRNAs (miR-4484, miR-4497, miR-4530, miR-3665, miR-4455, and miR-4443) exhibited significant fold changes in expression levels (*P* < 0.05).

To confirm the changes of miRNA expression levels induced by CVA16 and EV71 infections, six miRNAs were selected from the miRNA pool regulated by both viruses for expression analysis by qRT-PCR. As shown in Figure 2, miR-4484, miR-4497, miR-4530, and miR-3665 showed significant fold increase in expression levels, whereas miR-4455 and miR-4443 were markedly downregulated upon CVA16 and EV71 infections. These results were consistent with those obtained by miRNA microarray analysis.

### 3.3. miRNA Target Gene Prediction

To further unveil the possible molecular mechanisms underlying HFMD-related virus infection, comprehensive target gene prediction was performed based on the 27 differentially expressed miRNAs (15 upregulated and 12 downregulated, Table 2) from CVA16 and EV17 commonly regulated miRNA pool using the TargetScan and miRanda predicting procedures. Thousands of potential target genes of these 27 miRNAs were successfully predicted simultaneously by both algorithms following the corresponding mapping rules (Supplementary Table S1 in Supplementary Material available online at http://dx.doi.org/10.1155/2016/4302470). Further analysis revealed that each of the differentially expressed miRNAs could target a diversity of genes, while a specific gene could be also targeted by multiple miRNAs. Because immune responses are usually involved in the process of viral infection, many immune-related genes such as CD40 ligand (CD40LG), interleukin 6 receptor (IL-6R), interferon regulatory factor 4 (IRF4), and toll-like receptor 10 (TLR10) were predicted, all of which might participate in host antiviral responses against CVA16 and EV71.

### 3.4. GO Annotation and Enrichment Analysis

**Figure 3:**
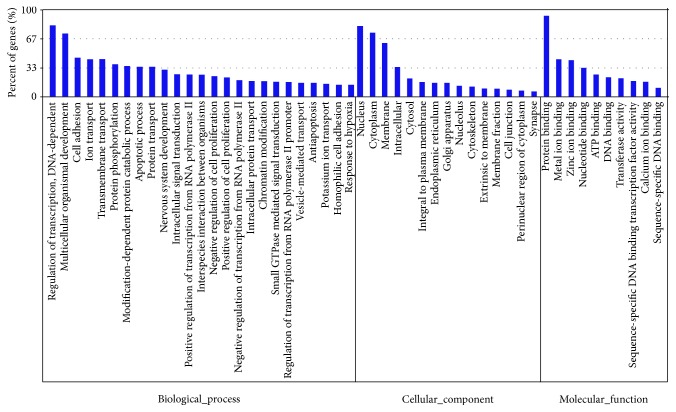
The target genes were classified to 3 main GO categories. *Y*-axis represented the percentage of genes in each enriched GO term.

A great number of genes could be probably regulated by CVA16- and EV71-associated miRNAs, whereas the biological characteristics of these genes were largely unknown. We therefore used GO database to perform overall analysis of target gene functions. GO is a standard classified system to describe the molecular function, cellular component, and biological process of a certain gene. The target genes were annotated with corresponding GO terms in Supplementary Table S1 and then subjected to enrichment analysis. The top 5 enriched terms in each GO category for the target genes were listed in Table 3. Meanwhile, the percentage of genes involved in each enriched term was shown in Figure 3.

### 3.5. KEGG Annotation and Enrichment Analysis

KEGG is a public database to describe the biological pathway in which gene is involved. Similarly, we assigned the miRNA target genes to KEGG pathways (Supplementary Table S1) and found that 54 of the pathways were significantly enriched (*P* < 0.01, Table 4 and Supplementary Table S2). More than 150 target genes were related to pathways in cancer (ko05200), MAPK signaling pathway (ko04010), and focal adhesion (ko04510). Wnt signaling pathway (ko04310), TGF-beta signaling pathway (ko04350), and mTOR signaling pathway (ko04150) were also among the enriched pathways of the target genes, reflecting complicated biological events induced by virus infection.

## 4. Discussion

At present, HFMD is still a global public health concern, especially in China. CVA16 and EV71 infections are responsible for most cases of HFMD. The viral pathogenesis, however, remains largely unknown to date. Emerging studies have highlighted the implication of miRNAs as critical effectors in complicated virus-host interaction networks [19–23]. Although sporadic reports revealed the association of a single host miRNA molecule with EV71 infection [24, 25], overall expression alterations of cellular miRNAs upon the infection of HFMD-associated pathogens need to be elucidated. The purpose of our study is to systematically identify the miRNAs involved in CVA16 and EV71 infections as well as investigate their physiological functions by bioinformatic analysis, thus promoting better understanding of HFMD pathological condition.

Microarray assays revealed the miRNA expression profiles in CVA16- and EV71-infected cells. The expression levels of a total of 27 common miRNAs (15 upregulated and 12 downregulated) were found to be significantly changed during the infection process of both viruses, suggesting that these 27 miRNAs might play important roles in the replication course and/or pathogenesis of two enteroviruses. One of the 27 differentially expressed miRNAs identified in our study, miR-1246, was increased by 3.95-fold at the expression level in EV71-infected cells. This finding was consistent with a previous study by Xu and colleagues [30]. They also found the EV71-mediated upregulation of miR-1246 in infected cells and further unveiled that miR-1246 suppressed the expression of disc-large homolog 3 (DLG3) gene by directly binding the 3′-UTR sequence, which contributed to the neurological disorders induced by EV71 infection. Although the other altered miRNAs have not shown a relationship with the infection of two enteroviruses, some of them seemed to be implicated in molecular mechanisms of host cell interactions with other viruses. For instance, respiratory syncytial virus (RSV), mainly infecting infants and children like HFMD-associated pathogens, induced unique patterns of miRNA expression with specific upregulation of miR-30b in epithelial cells through the NF-*κ*B signaling pathway, suggesting a possible host defense against RSV by the miRNA response [31]. Similarly, miR-30b was also shown to be tightly associated with chronic HBV and HCV infection [32, 33]. In addition, changes in abundance of circulating miR-320c and miR-494 were also observed in serum of HCV infected individuals [34, 35]. At this moment, it is still unknown about the roles of these differentially expressed miRNAs on CVA16 and EV71 viral infection process. Functional studies need to be done with the use of corresponding miRNA mimics or inhibitors in the near future to further investigate their effects on viral replication as well as related molecular mechanisms. Since the 27 miRNAs simultaneously participated in both CVA16 and EV71 interactions with host cells, it is possible that one or two of them could serve as potential candidates for antiviral therapy of HFMD.

It should be noted that host cells exhibited some differences at the miRNA level in response to CVA16 and EV71 infections, although both viruses belong to members of the genus* Enterovirus*. CVA16 specifically led to significant expression changes of 15 miRNAs (9 upregulated and 6 downregulated), while the expression levels of 5 miRNAs (2 upregulated and 3 downregulated) were markedly altered only in EV71-infected cells (Table 2). This may reflect distinct pathological mechanisms underlying the progression of CVA16 or EV71 infection. Indeed, compared with CVA16, acute EV71 infection can cause HFMD frequently followed by serious nervous system diseases, including encephalitis, aseptic meningitis, and acute flaccid paralysis, thus possessing higher mortality. Immunocytochemical staining analysis revealed the existence of viral antigen in neurons, demonstrating the possibility that EV71 actively propagated in nerve cells [36, 37]. Therefore, it is not surprising that miRNAs may play an essential role in EV71-induced neuropathogenesis. Whether or not the EV71-specific miRNAs (miR-26b, 20b, 574-3p, 1280, and 22) identified in our study are associated with neurological disorders caused by viral invasion will require further investigation. Previous studies have reported that miR-26b could regulate neuronal development and plasticity by direct targeting of brain-derived neurotrophic factor (BDNF) [38]. Overexpression of miR-26b led to aberrant cell cycle entry, tau hyperphosphorylation, and apoptosis in postmitotic neurons, which might contribute to the Alzheimer's disease (AD) neuronal pathology [39]. Meanwhile, miR-22 has been proved to be a potentially neuroprotective miRNA. Its diminished expression was also observed in Huntington's disease (HD) and elevated cellular levels inhibited neurodegeneration in* in vitro* models through a reduction in caspase activation [40]. These previously elucidated mechanisms will provide guidance for future studies of the involvement of miRNAs in neuropathogenesis caused by EV71 infection.

The EV71-mediated miRNA expression profile identified in present study displayed a little difference from that performed in previous report by Xu et al. [30]. This discrepancy might be largely due to different cell types used in viral infection between studies. RD cells, as the most sensitive cell line, have been most frequently applied for enteroviral infections in a variety of studies, whereas Xu and colleagues used SH-SY5Y cells, a human neuroblastoma cell line, to examine the host miRNA response to EV71 infection in nerve cells with the purpose of revealing miRNA-mediated neurological pathogenesis. It is well known that miRNAs have unique tissue cell-specific expression patterns. Each human tissue is characterized by a specific set of miRNAs which may constitute an innate characteristic of that tissue [11, 41]. Moreover, an increasing number of experimental researches have demonstrated that some miRNAs have the potential to modulate the* in vivo* tissue tropism of multiple viruses [42–44]. So it can be plausible that there is substantial heterogeneity in miRNA expression profile if different tissue cell models were used for the infection of certain pathogen, which depends on further investigation in the near future. Despite this fact, our results confirmed and expanded the findings from a recent study [45] that several cellular miRNA molecules (miR-3665, 4443, 4497, 4530, and 494) were significantly changed not only by EV71 but also by CVA16 viral infection.

In general, mature miRNA population functions as regulators at the posttranscriptional level by directly targeting 3′-UTR regions of mRNAs. Identification of their target genes is therefore a key step to understand the physiological and pathological functions of miRNAs. Target prediction algorithms have been developed as a straightforward and powerful tool to search for the putative miRNA-regulated genes based on seed sequence matching principle. Jointly using two independent target predicting procedures, a great number of target genes of 27 miRNAs coassociated with CVA16 and EV71 infection were obtained. Many of them were related to interleukin, chemokine, interferon, and toll-like receptor, all of which had the ability to mediate host innate and adaptive immune responses against infection of pathogens [46–49]. GO function annotation and enrichment analysis of the target genes according to three categories of biological process, cellular component, and molecular function demonstrated that viral infection might lead to a wide range of regulatory events in host system. Subsequently, KEGG pathway analysis of these putative target genes indicated involvement of Wnt signaling pathway, MAPK signaling pathway, TGF-beta signaling pathway, and mTOR signaling pathway, which have been shown to be important during the infection processes of some other viruses [50–53]. The bioinformatic analysis described above provided a preliminary dataset for additional screening work to characterize the link between specific miRNAs and target genes during host responses to infection of HFMD-associated pathogens.

Taken together, our present study showed that CVA16 and EV71 infections did alter the miRNA expression patterns in host cells with common and specific miRNAs expression changes. Furthermore, systematic bioinformatic analysis provided a comprehensive overview about the relationship between miRNAs and their targets, thus promoting a deeper understanding of viral pathogenesis as well as facilitating the possible development of a miRNA-based therapeutic approach for the prevention and control of HFMD.

## Supplementary Material

Supplementary Table S1: The list of target genes of 27 common differentially expressed miRNAs predicted by TargetScan and MiRanda and corresponding GO/KEGG annotation. Supplementary Table S2: The KEGG pathways enriched in the target genes of 27 common differentially expressed miRNAs.

## Figures and Tables

**Table 1:** Specific primer sequences for miRNA expression validation by quantitative RT-PCR.

Primer	Sequence (5′-3′)
miR-4484	AAAAGGCGGGAGAAGCCCCAAA
miR-4497	ATTCTCCGGGACGGCTGGGCA
miR-4530	ATTCCCAGCAGGACGGGAGCGAA
miR-3665	ATTAGCAGGTGCGGGGCGGCGAA
miR-4455	CCAGGGTGTGTGTGTTTTTAA
miR-4443	TTGGAGGCGTGGGTTTT
U6F	CGCTTCGGCAGCACATATAC
U6R	ACGAATTTGCGTGTCATCCT

**Table 2:** The differentially expressed miRNAs upon CVA16 and EV71 infections.

	Upregulated		Downregulated	
	miRNA	Fold change	miRNA	Fold change
CVA16 and EV71 common	miR-4484	5.47/3.99^*∗*^	miR-4455	−3.43/−2.13
	miR-4497	4.38/1.54	miR-1260b	−2.72/−1.84
	miR-4530	4.36/2.05	miR-4324	−2.48/−2.44
	miR-1246	1.89/3.95	miR-720	−2.28/−2.07
	miR-494	1.76/3.11	miR-4534	−2.20/−1.53
	miR-3665	2.94/2.02	miR-107	−2.02/−1.69
	miR-3935	2.89/2.62	miR-4443	−1.83/−1.88
	miR-3591-3p	1.95/2.78	miR-320a	−1.63/−1.79
	miR-5096	2.40/1.82	miR-103a	−1.78/−1.57
	miR-4277	2.16/1.52	miR-320c	−1.56/−1.65
	miR-4734	2.04/1.85	miR-23c	−1.61/−1.54
	miR-19b	2.00/1.70	miR-320b	−1.59/−1.55
	miR-181d	1.56/1.87		
	miR-30b	1.64/1.83		
	miR-4687-3p	1.64/1.66		

CVA16-specific	miR-4508	2.99	miR-4668-5p	−2.49
	miR-638	2.95	let-7b	−1.65
	miR-762	2.32	miR-320d	−1.55
	miR-4466	2.16	miR-151-5p	−1.53
	miR-4787-5p	2.03	miR-4739	−1.51
	miR-3196	1.98	miR-320e	−1.50
	miR-3960	1.97		
	miR-4516	1.86		
	miR-3656	1.82		

EV71-specific	miR-26b	1.82	miR-574-3p	−1.59
	miR-20b	1.51	miR-1280	−1.54
			miR-22	−1.52

^*∗*^The fold change of a miRNA regulated by CVA16/EV71 infection.

**Table 3:** The 5 most enriched terms in each GO category for the target genes of 27 differentially expressed miRNAs.

GO ID	GO term	Gene number	*P* value
*Biological process*			
GO: 0019941	Modification-dependent protein catabolic process	292	2.12*E* − 10
GO: 0007399	Nervous system development	259	4.10*E* − 09
GO: 0006355	Regulation of transcription, DNA-dependent	760	4.90*E* − 09
GO: 0007242	Intracellular signal transduction	210	1.08*E* − 07
GO: 0006468	Protein phosphorylation	315	1.50*E* − 06
*Cellular component*			
GO: 0005634	Nucleus	3367	1.23*E* − 20
GO: 0005737	Cytoplasm	3041	1.52*E* − 17
GO: 0005794	Golgi apparatus	603	4.61*E* − 13
GO: 0005622	Intracellular	1355	9.80*E* − 13
GO: 0016020	Membrane	2561	2.50*E* − 10
*Molecular function*			
GO: 0005515	Protein binding	3825	9.16*E* − 28
GO: 0008270	Zinc ion binding	1689	1.03*E* − 25
GO: 0046872	Metal ion binding	1759	5.63*E* − 24
GO: 0003700	Sequence-specific DNA binding transcription factor activity	672	1.01*E* − 09
GO: 0000166	Nucleotide binding	1325	1.51*E* − 07

**Table 4:** The 15 KEGG pathways most enriched in the target genes of 27 differentially expressed miRNAs.

Pathway ID	Pathway description	Gene number	*P* value
ko05200	Pathways in cancer	256	1.18*E* − 11
ko04722	Neurotrophin signaling pathway	106	3.31*E* − 09
ko04012	ErbB signaling pathway	75	8.40*E* − 08
ko04310	Wnt signaling pathway	121	8.74*E* − 08
ko04144	Endocytosis	145	2.31*E* − 07
ko04910	Insulin signaling pathway	109	7.69*E* − 07
ko04510	Focal adhesion	153	8.55*E* − 07
ko05214	Glioma	57	9.27*E* − 07
ko05211	Renal cell carcinoma	60	2.64*E* − 06
ko04720	Long-term potentiation	60	2.64*E* − 06
ko04916	Melanogenesis	83	3.03*E* − 06
ko04010	MAPK signaling pathway	199	4.31*E* − 06
ko04666	Fc gamma R-mediated phagocytosis	79	4.93*E* − 06
ko04514	Cell adhesion molecules (CAMs)	104	1.14*E* − 05
ko04070	Phosphatidylinositol signaling system	63	1.52*E* − 05
