# Supplementary material for: Systematic Identification and Bioinformatic Analysis of MicroRNAs in Response to Infections of Coxsackievirus A16 and Enterovirus 71
Source: Biomed Res Int. 2016 Oct 24;2016:4302470. doi: 10.1155/2016/4302470 (PMC5098103; doi:10.1155/2016/4302470)
Supplement: Supplementary file 2 [file 4302470.f2.doc]

**Supplementary Table S2.** The other KEGG pathways enriched in the target genes of 27 differentially expressed miRNAs.

| **Pathway ID** | **Pathway description** | **Gene number** | ***P* value** |
| --- | --- | --- | --- |
| ko05215 | Prostate cancer | 72 | 2.06E-05 |
| ko05220 | Chronic myeloid leukemia | 62 | 2.13E-05 |
| ko04210 | Apoptosis | 71 | 2.81E-05 |
| ko05212 | Pancreatic cancer | 59 | 5.67E-05 |
| ko05210 | Colorectal cancer | 68 | 6.98E-05 |
| ko04360 | Axon guidance | 98 | 9.52E-05 |
| ko05223 | Non-small cell lung cancer | 45 | 2.06E-04 |
| ko04920 | Adipocytokine signaling pathway | 54 | 2.74E-04 |
| ko04115 | p53 signaling pathway | 55 | 4.14E-04 |
| ko04020 | Calcium signaling pathway | 128 | 5.93E-04 |
| ko04350 | TGF-beta signaling pathway | 67 | 6.26E-04 |
| ko05222 | Small cell lung cancer | 66 | 8.13E-04 |
| ko04730 | Long-term depression | 56 | 1.14E-03 |
| ko05218 | Melanoma | 55 | 1.49E-03 |
| ko04520 | Adherens junction | 59 | 1.64E-03 |
| ko00601 | Glycosphingolipid biosynthesis - lacto and neolacto series | 23 | 1.64E-03 |
| ko04530 | Tight junction | 97 | 1.89E-03 |
| ko04912 | GnRH signaling pathway | 75 | 1.94E-03 |
| ko04340 | Hedgehog signaling pathway | 44 | 2.68E-03 |
| ko04660 | T cell receptor signaling pathway | 79 | 3.04E-03 |
| ko04150 | mTOR signaling pathway | 41 | 3.32E-03 |
| ko04120 | Ubiquitin mediated proteolysis | 98 | 3.32E-03 |
| ko04810 | Regulation of actin cytoskeleton | 149 | 3.82E-03 |
| ko00512 | O-Glycan biosynthesis | 26 | 4.12E-03 |
| ko04062 | Chemokine signaling pathway | 132 | 4.25E-03 |
| ko05412 | Arrhythmogenic right ventricular cardiomyopathy (ARVC) | 57 | 4.56E-03 |
| ko04370 | VEGF signaling pathway | 57 | 4.56E-03 |
| ko00562 | Inositol phosphate metabolism | 42 | 4.70E-03 |
| ko04630 | Jak-STAT signaling pathway | 109 | 4.95E-03 |
| ko04914 | Progesterone-mediated oocyte maturation | 76 | 5.69E-03 |
| ko04540 | Gap junction | 66 | 5.90E-03 |
| ko04142 | Lysosome | 83 | 6.56E-03 |
| ko05414 | Dilated cardiomyopathy | 67 | 7.28E-03 |
| ko05410 | Hypertrophic cardiomyopathy (HCM) | 65 | 7.32E-03 |
| ko05213 | Endometrial cancer | 40 | 8.09E-03 |
| ko05014 | Amyotrophic lateral sclerosis (ALS) | 42 | 8.39E-03 |
| ko05217 | Basal cell carcinoma | 42 | 8.39E-03 |
| ko05219 | Bladder cancer | 33 | 9.11E-03 |
| ko00564 | Glycerophospholipid metabolism | 52 | 9.17E-03 |
